# Identifying Factors of Organoid Establishment in Pancreatic Cancer: A Prospective Observational Study

**DOI:** 10.1002/cam4.71490

**Published:** 2025-12-30

**Authors:** Katharina Wansch, François Schneider, Florian Dölvers, Anna Kühn, Mihnea P. Dragomir, Maria Joosten, Georg Hilfenhaus, Loredana Vecchione, Matthäus Felsenstein, Markus Lerchbaumer, Christian Jürgensen, Marcus Bahra, Gregor Duwe, Reinhold Schäfer, Sebastian Stintzing, Ulrich Keilholz, Christopher C. M. Neumann, Uwe Pelzer

**Affiliations:** ^1^ Department of Hematology, Oncology and Tumor Immunology Charité‐Universitätsmedizin Berlin, Freie Universität Berlin, Humboldt‐Universität zu Berlin, Berlin Institute of Health Berlin Germany; ^2^ Department of Pathology Charité‐Universitätsmedizin Berlin, Freie Universität Berlin, Humboldt‐Universität zu Berlin, Berlin Institute of Health Berlin Germany; ^3^ German Cancer Consortium (DKTK) Partner Site Berlin and German Cancer Research Center (DKFZ) Heidelberg Germany; ^4^ Berlin Institute of Health (BIH) Berlin Germany; ^5^ Department of Surgery|CCM|CVK Charité‐Universitätsmedizin Berlin, Freie Universität Berlin, Humboldt‐Universität zu Berlin, Berlin Institute of Health Berlin Germany; ^6^ Department of Radiology Charité‐Universitätsmedizin Berlin, Freie Universität Berlin, Humboldt‐Universität zu Berlin, Berlin Institute of Health Berlin Germany; ^7^ Department of Hepatology and Gastroenterology Charité‐Universitätsmedizin Berlin, Freie Universität Berlin, Humboldt‐Universität zu Berlin, Berlin Institute of Health Berlin Germany; ^8^ Department of Surgical Oncology and Robotics Krankenhaus Waldfriede, Lehrkrankenhaus der Charité Berlin Germany; ^9^ Department of Urology and Pediatric Urology University Medical Center Johannes Gutenberg University Mainz Germany; ^10^ Charité Comprehensive Cancer Center Charité‐Universitätsmedizin Berlin Berlin Germany

**Keywords:** functional precision medicine, pancreatic ductal adenocarcinoma, patient‐derived organoids

## Abstract

**Background:**

Patient‐derived organoid (PDO) models have emerged as critical tools in pancreatic ductal adenocarcinoma (PDAC) research and are used as surrogates for studying the individual's tumor biology. Still, PDO‐based concepts for direct clinical application remain challenging. In this prospective observational trial (OrgaPanCCC‐01), we aim to address clinical feasibility, identify predictive factors for PDO establishment, and assess the prognostic potential of PDO establishment for patient's survival.

**Methods:**

Samples for PDO generation were prospectively collected via endoscopy, surgery, and transcutaneous punch biopsy, or from ascites. Patients were followed up for a median time of 14.6 months. We evaluated the clinical feasibility by determining the PDO establishment rate and the time required for establishment. Uni‐ and multivariate analyses were performed to examine the effect of clinical and sample characteristics on PDO establishment. For the predictive and prognostic potential, PDO establishment was correlated to the patients' disease‐free (DFS), progression‐free (PFS) and overall survival (OS).

**Results:**

Between 2021 and 2023, 75 patients were enrolled with radiologically suspected PDAC at the Charité Universitätsmedizin Berlin and at the Waldfriede Krankenhaus Berlin. PDAC was confirmed in 62 patients (83%). PDO establishment was achieved in 58% (*n* = 36/62) of patients within a median of 28 days, supporting the feasibility of clinical implementation. In the uni‐ and multivariate analysis, samples from metastatic sites (*p* = 0.04) and higher CA19‐9 levels (*p* = 0.03) were found to be positively correlated with PDO growth. Patients without PDO growth tended to have longer PFS (*p* = 0.32), whereas no statistically significant correlation was observed between PDO growth and OS.

**Conclusion:**

In this prospective observational trial, we show that PDO generation is feasible at a success rate of 58% within a clinically reasonable time frame of 6 weeks. The efficacy of PDO establishment depended on sample site, with metastatic samples showing higher establishment rates. Higher CA 19‐9 levels were positively correlated with PDO growth. Successful PDO establishment did not have a prognostic value for OS. Overall, our findings underline the great potential of PDO‐based precision medicine approaches, which should further be evaluated in prospective interventional translational trials.

## Graphical abstract

Systematic overview of the prospective observational trial OrgaPanCCC‐01.

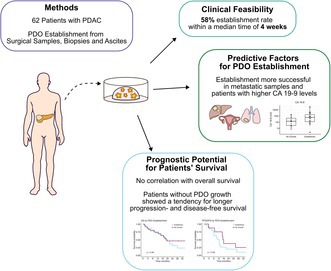

**Abbreviations**
ACC: acinar cell carcinoma
CAR: CRP‐albumin ratio
CCC: cholangiocellular carcinoma
DFS: disease‐free survival
DLBCL: diffuse large b‐cell lymphoma
FNB: fine needle biopsy
IBI: inflammatory benchmark index
LMR: lymphocyte‐monocyte ratio
MPC: medullary pancreatic cancer
NET: neuroendocrine tumor
NLR: neutrophil‐lymphocyte ratio
OS: overall survival
PAC: periampullary cancer
PDAC: pancreatic ductal adenocarcinoma
PDO: patient‐derived organoid
PDX: patient‐derived xenograft
PFS: progression‐free survival
PLR: platelet‐lymphocyte ratio
RCC: renal cell carcinoma

## 1 Introduction

Pancreatic adenocarcinoma (PDAC) is among the most lethal cancer entities with a highly limited 5‐year OS rate of 11% [1]. Targeted inhibitors are only available for approximately 10% of PDAC patients in routine clinical practice, whereas chemotherapy remains the systemic treatment of choice for all other patients [2, 3]. However, treatment success is limited since PDAC is characterized by an aggressive tumor biology and high levels of therapy resistance [3]. In order to identify more effective treatment options and to study the dynamic tumor biology, patient‐derived organoids (PDOs) experienced remarkable attention in the last few years [4, 5]. These three‐dimensional cell culture models derived from stem‐like cells were first described by Sato et al. [6] and have since revolutionized cancer research [4, 7, 8, 9]. Their organ‐like structure more accurately represents the tumor's biology than conventional 2D cell culture models [4, 10, 11]. Today, organoids are established tools for examining tumor cell plasticity [5], inducing therapy resistance [12], identifying molecular alterations in tumor cells [4] and predicting therapy response [4, 13, 14, 15]. As a consequence, the FDA has recently announced a new strategy for drug approval, which includes the replacement of animal testing by in vitro PDO methodology [16]. In PDAC, several prospective studies have evaluated the potential of PDOs for therapy prediction, altogether showing high correlation between PDO testing and patients' clinical response [17, 18, 19, 20, 21]. Yet, more data on factors affecting clinical feasibility, predictive factors for PDO establishment and the prognostic potential of PDO establishment for patients' survival need to be collected. To ensure the clinical feasibility of PDO‐based precision medicine, two fundamental requirements must be met. First, PDOs need to be established for almost all patients to ensure broad applicability. Secondly, establishment must be completed within a time frame that aligns with clinical decision‐making. To realize PDO‐based pharmacotyping effectively guiding clinical treatments, pharmacotyping information must be made available prior to or during therapy initiation. For patients with resectable PDAC, therapy is started within the first 12 weeks after surgery [22, 23, 24]. Patients with metastatic disease typically start treatment shortly after histological verification. Thus, PDO pharmacotyping results should be available within a maximum period of 12 weeks, enabling a PDO‐based therapy initiation for resected patients and an early change in the chemotherapeutic regimen for metastatic patients. Various studies have shown that PDO establishment is feasible within this period [18, 19, 21]. Establishment rates, on the other hand, vary widely, ranging from 41% to 80% [17, 18, 19, 20, 21, 25, 26]. In order to understand this variability and to improve establishment rates, it is critical to gain a comprehensive understanding of factors influencing PDO growth. So far, the only consistent variable affecting PDO establishment is proven to be high tumor cellularity [13, 18, 25, 27]. Beyond this, the sample type and patients' pretreatment can influence PDO growth, but these findings have been inconsistent and even in some studies contradictory [13, 17, 18, 19, 20, 25]. For instance, while samples from fine needle biopsies (FNBs) exhibited the lowest establishment rates in a study by Grossman et al. with 11% [18], the opposite was the case in the cohort of Matsumoto et al. with 80% [25]. Other working groups identified additional factors. These included short processing times [28] and the number of live cells after tumor digestion [25] which positively correlated with PDO growth. Overall, a more thorough investigation is necessary to determine the reliability of these initial findings. An additional emerging question in PDO research concerns the use of PDO growth as a prognostic marker for survival. This correlation has been shown for patient‐derived xenograft (PDX) engraftment in PDAC, head‐ and neck cancer and breast cancer [29, 30, 31]. However, there is little evidence on this for PDAC PDOs. Two recent studies [27, 32] examined the relationship between PDO growth and patient survival. While one study found a significant association, the second study could not replicate these results [27, 32].

In summary, there is limited evidence, not only on factors impacting PDO growth, but also its subsequent prognostic value for the individual's survival. The aim of this prospective observational study was to assess whether PDO technology is clinically feasible, to identify the predictive clinical parameters for PDO establishment and to assess the prognostic potential of PDO establishment for patients' survival.

## 2 Methods

### 2.1 Patient Inclusion

Samples were collected from November 2021 to September 2023 from patients with suspected PDAC, who underwent surgery or diagnostic procedures. We recruited patients at two pancreatic cancer centers, the Charité Universitätsmedizin Berlin and the Krankenhaus Waldfriede Berlin‐Zehlendorf. The work was performed in alignment with the ethics approval (EA1/157/21) and all patients gave informed consent.

### 2.2 Patient Follow Up and Clinical Information

Clinical data and laboratory parameters were obtained before the patients received any diagnostic or curative procedure. Laboratory parameters were part of standard clinical practice. Patients were followed up for a minimum period of 6 months following study enrollment. Median follow‐up time was 14.6 months. The last follow‐up was completed in May 2025. OS was assessed for all patients. For patients with resectable disease, DFS was defined as the time from resection to the time of relapse. For patients with metastatic disease, PFS was defined as the time from therapy initiation to the time of tumor progression. Patients who deceased intraoperatively or from postoperative complications within the first 2 weeks were excluded from survival analyses. All patients were treated according to standard clinical practice.

### 2.3 Sample Collection

For patients with resectable disease, tissue was obtained from surgical resection samples of the primary tumor. For patients with metastatic disease, samples from the primary tumor were obtained by endoscopic ultrasound guided FNB and from surgical resections in a palliative setting. Samples from the metastases were obtained from palliative surgeries, transcutaneous punch biopsies, and ascites.

The tumor tissue was transported in RPMI‐1640 media (Thermo Fisher Scientific, Waltham, MA, USA) containing 1% Gentamicin (Thermo Fisher Scientific, Waltham, MA, USA) and 1% Penicilin/Streptomycin (Thermo Fisher Scientific, Waltham, MA, USA). During the transportation, the tumor tissue was cooled. All samples were processed immediately upon arrival in the laboratory. The time from sample acquisition to sample processing ranged from 15 to 45 min for FNB samples, punch biopsy samples, and ascites. For surgical samples, the time from sample acquisition to tissue processing ranged from 1 to 2 h.

### 2.4 Establishment of Organoids

After sample collection, tumor samples were cut into small fragments and enzymatically digested to obtain a cell pellet for organoid formation. For enzymatic digestion of surgical samples and biopsies, a solution containing 100 μg/mL DNAse I (VWR, Radnor, PA, USA), 100 μg/mL Dispase (STEMCELLTechnologies, Vancouver, Canada), 125 μg/mL Collagenase II (Sigma‐Aldrich, Merck, Darmstadt, Germany), 1:2000 Rock‐Inhibitor (Abmole Bioscience, Houston, TX, USA) and 1:200 Amphotericin B (Sigma‐Aldrich, Merck, Darmstadt, Germany) was used. Samples were digested at 37°C, digestion time ranged from 5 to 15 min for FNBs and to 3 h for surgical specimens. Cells from ascites were isolated by centrifuging the malignant effusion at 300 rcf to obtain cell pellets. Red blood cell lysis (Miltenyi Biotec, Bergisch‐Gladbach, Germany) was performed for 5 to 10 min if necessary. For ascites samples, immune cells were further depleted by magnetic separation using CD45+ magnetic microbeads (Miltenyi Biotec, Bergisch‐Gladbach, Germany) with a MACS Separator (Miltenyi Biotec, Bergisch‐Gladbach, Germany) according to the manufacturer's protocol. The cell pellets were embedded in Corning Matrigel Growth Factor Reduced (GFR) Basement Membrane Matrix (Corning, Corning, NY, USA) or Cultrex Reduced Growth Factor BME, Type 2 (R&D Systems, Minneapolis, MN, USA). Medium as first described by Broutier, Andersson‐Rolf, Hindley, Boj, Clevers, Koo and Huch [33] was added. For the first 7 days of culturing, the medium was supplemented with Amphotericin B at a 1:200 dilution. To prevent mycoplasma contamination, mycoplasma tests were performed using a Mycoplasma detection kit (Applied biological Materials, Richmond, Canada). All PDOs were tested negative for mycoplasma contamination. PDOs were split when reaching a size > 200 μm. Splitting was performed mechanically and enzymatically using TrypLE Express (Thermo Fisher Scientific, Waltham, MA, USA). Based on previous PDO studies, organoids were defined as established after reaching culture passage three [19, 21, 34]. After successful establishment, PDOs were further expanded for functional testing or frozen and stored in liquid nitrogen.

### 2.5 Histopathological Characterization

Histopathological characterization was conducted in a sub‐cohort of 20 FFPE samples by a reference pathologist. These included 10 samples of successful PDO growth. *K*‐nearest neighbors' (*k* = 6) analysis was performed for all patient‐based parameters of this study to match the 10 established PDOs to 10 patients from which PDO growth was not successful. Histopathological characterization included H&E staining of FFPE blocks to assess tumor cellularity and the percentage of tumor necrosis. Immunohistochemical staining was performed with the BenchMark XT immunostainer (Ventana Medical Systems, Tucson, AZ), using an anti‐Ki‐67 (MIB‐1, Dako, 1:50) and a GATA6 antibody (R&D Systems, AF1700, polyclonal, 1:100).

### 2.6 Statistical Analysis

The statistical analysis was performed with R/R‐Studio (version 4.4.1, R Core Team 2024). For descriptive statistics, the Table 1 package was used. Plots were generated using the ggplot2 and ggbreak [35] packages. For all continuous variables, outliers were identified using the ROUT method in GraphPadPrism (Version 10.6.0) and the IQR method in R/R‐Studio. Outliers were subsequently excluded for further analyses. To perform the uni‐ and multivariate analysis, a binomial regression was used. For the multivariate analysis, a limited number of three variables were analyzed to avoid overfitting. The selection of these variables was based on clinical relevance, previously published studies and the significance in the univariate analysis [36, 37, 38]. If causal inference was of interest, confounders were identified for each exposure‐outcome pair. Confounder identification was performed using DAGs generated in the dagitty R package [39]. Adjustment for these confounders was then performed using a binomial regression model. For both the uni‐ and multivariate analysis, continuous variables were dichotomized using previously reported cut‐off values [40]. Cut‐offs were 65 years for age, 1.35/nL for lymphocytes, 235/nL for platelets, 0.6/nL for monocytes, 5/nL for neutrophils, 5 mg/L for CRP, 500 U/mL for CA 19‐9, 4.0 for neutrophil‐lymphocyte ratio (NLR), 0.4 for CRP‐albumin ratio (CAR), 1.6 for lymphocyte‐monocyte ratio (LMR), 180 for platelet‐lymphocyte ratio (PLR) and 30 for the inflammatory benchmark index (IBI), 30 mg/L for Albumin, 1.1 mg/dL for Bilirubin, 18.5 kg/m^2^ for BMI, 40 U/L for GGT, 10 g/dL for Hemoglobin, 7.2 μg/L for CEA, 250 U/L for LDH. A cox proportional hazards regression was used for survival analyses with the log‐rank test used to determine statistical significance. This was conducted using the survminer and survival packages in R. Results were considered statistically significant if the *p*‐value was below 0.05.

**TABLE 1:** Overview of patient characteristics for PDAC patients.

	Overall	Established	No growth
	*N* = 62	*N* = 36 (%)	*N* = 26 (%)
Age			
Median [min–max]	69.0 [42.0–91.0]	66.0 [42.0–86.0]	69.5 [47.0–91.0]
Sex			
Female	40 (64.5)	26 (72.2)	14 (53.8)
Male	22 (35.5)	10 (27.8)	12 (46.2)
UICC stage			
I	9 (14.5)	5 (13.9)	4 (15.4)
II	20 (32.3)	10 (27.8)	10 (38.5)
III	9 (14.5)	4 (11.1)	5 (19.2)
IV	24 (38.7)	17 (47.2)	7 (26.9)
Pretreatment			
Untreated	52 (83.9)	30 (83.3)	22 (84.6)
Pretreated	10 (16.1)	6 (16.7)	4 (15.4)
GemNab	2 (3.2)	1 (2.8)	1 (3.8)
mFOLFIRINOX	6 (9.6)	5 (13.9)	1 (3.8)
Capecitabine	1 (1.6)	1 (2.8)	0 (0)
Irinotecan/5‐FU	2 (3.2)	0 (0)	2 (7.7)
Resection status			
Resectable	38 (61.3)	19 (52.8)	19 (73.1)
R0‐Resection	24 (38.7)	13 (36.1)	11 (42.3)
R1‐Resection	13 (21.0)	6 (16.7)	7 (26.9)
Unknown	1 (1.6)	0 (0)	1 (3.8)
Metastasized	24 (38.7)	17 (47.2)	7 (26.9)
Diabetes			
Yes	9 (14.5)	3 (8.3)	6 (23.1)
No	26 (41.9)	19 (52.8)	7 (26.9)
Unknown	27 (43.5)	14 (38.9)	13 (50.0)
BMI			
Median [min–max]	23.8 [14.7–32.0]	22.8 [14.7–32.0]	25.0 [19.5–31.9]
Missing	12 (19.4)	10 (27.8)	2 (7.7)
Patient survival			
Alive	27 (43.5)	14 (38.9)	13 (50.0)
Deceased	34 (54.8)	21 (58.3)	13 (50.0)
Unknown	1 (1.6)	1 (1.6)	0 (0)

## 3 Results

### 3.1 Characteristics of the Patient Cohort

**FIGURE 1:**
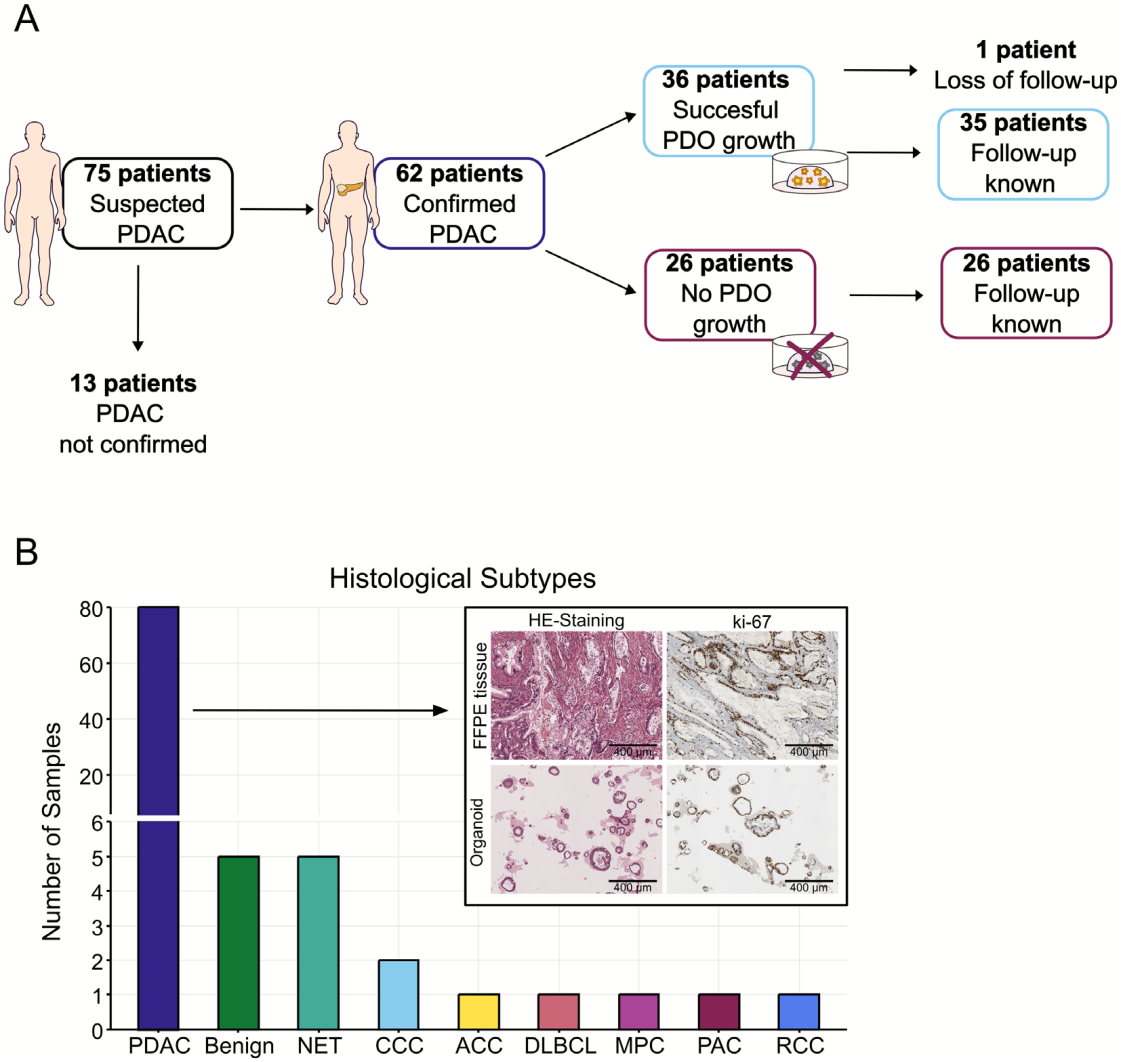
Inclusion of patients and histological subtypes of samples included for PDO‐establishment. (A) Number of patients included in each step of the study. (B) Number of samples per histological subtype. HE‐ and Ki‐67 staining are shown for FFPE tissue and PDOs of one exemplary patient, scale bars represent 400 μm. ACC, acinar cell carcinoma; CCC, cholangiocellular carcinoma; DLBCL, diffuse large b‐cell lymphoma; MPC, medullary pancreatic cancer; NET, neuroendocrine tumor; PAC, periampullary cancer; PDAC, pancreatic ductal adenocarcinoma; RCC, renal cell carcinoma.

During the course of 2 years (2021–2023), a total of 97 samples were collected from 75 patients with suspected PDAC (Figure 1A). The median age at the time of inclusion was 67 years (41–91 years). A minor gender disparity was documented (m:f 0.43:0.57). Most patients (64%) were included at a resectable stage (Table S1). The final histopathological report confirmed PDAC in 80 samples (82%) from 62 patients (Figure 1B). In the remaining samples, benign pancreatic tissue and neuroendocrine tumors were found most frequently (Figure 1B). These samples were excluded for further analyses. Within the cohort of PDAC patients, the majority (83.9%) were treatment‐naïve at the time of enrolment to the study. A small subset of 10 patients received treatment prior to sample acquisition. PDOs were successfully established for 36 patients (58%). From 61 of the 62 patients (98%), clinical follow‐up was attainable at a median follow‐up time of 14.6 months (5.3 to 45.5 months).

### 3.2 PDO Establishment Is Feasible Within a Clinically Reasonable Time Frame

**FIGURE 2:**
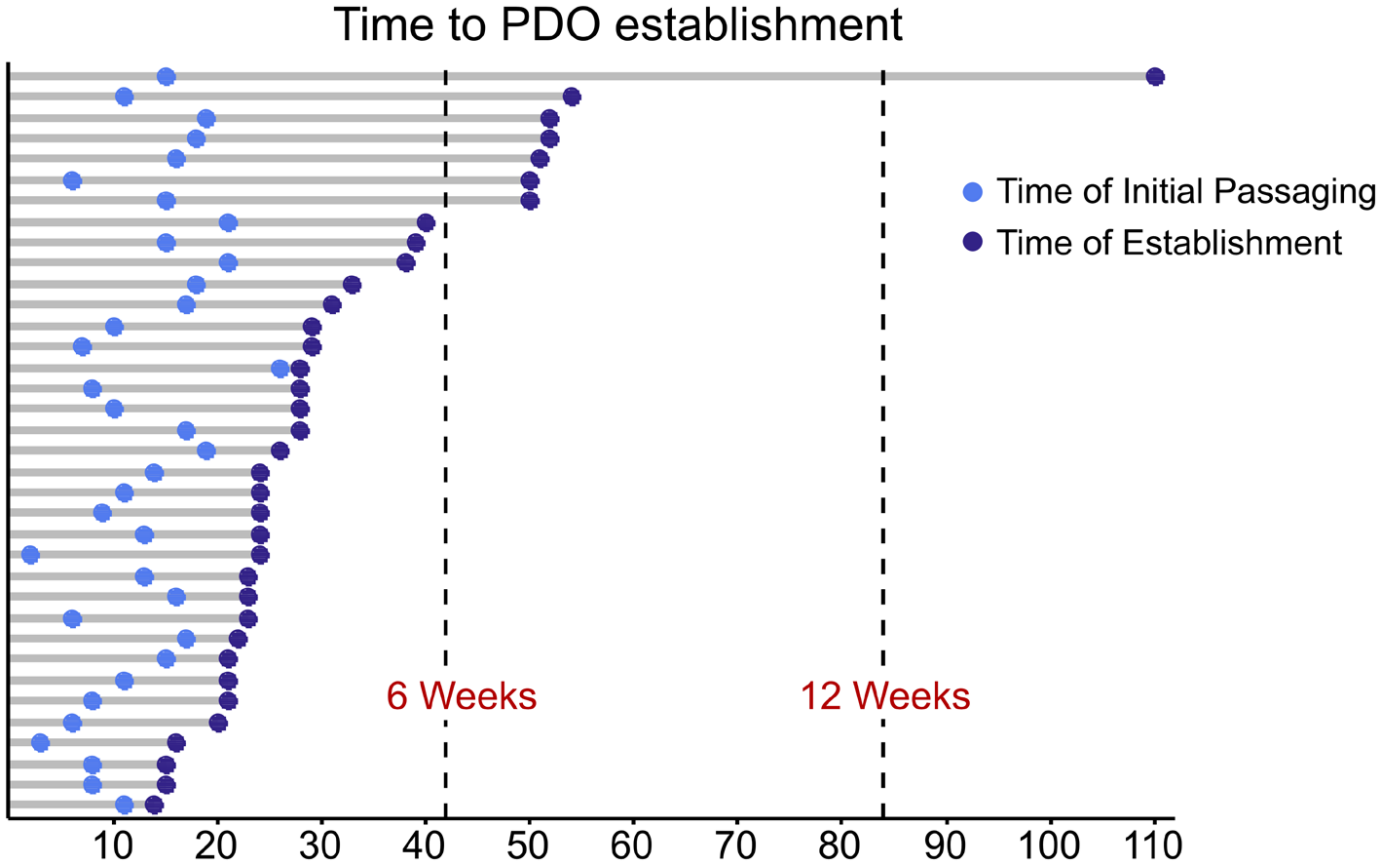
Time to PDO establishment for established PDO samples. Light blue dots symbolize the time after which PDOs could first be passaged. Dark blue dots show the time after which PDOs reached passage three. The vertical lines represent important clinical time frames for PDO establishment (6 and 12 weeks).

Overall, PDOs were established in 58% (*n* = 36/62) of PDAC patients within a clinically actionable time of 12 weeks. 80% of these samples were established within 6 weeks. PDOs were first passaged at a median of 14 days, with a median time to establishment of 28 days (Figure 2). In one case of a FNB, the establishing process took 110 days.

### 3.3 Assessment of Predictive Factors for PDO Growth

#### 3.3.1 Establishment Efficacy Varies by Sample Site and Sample Type

**FIGURE 3:**
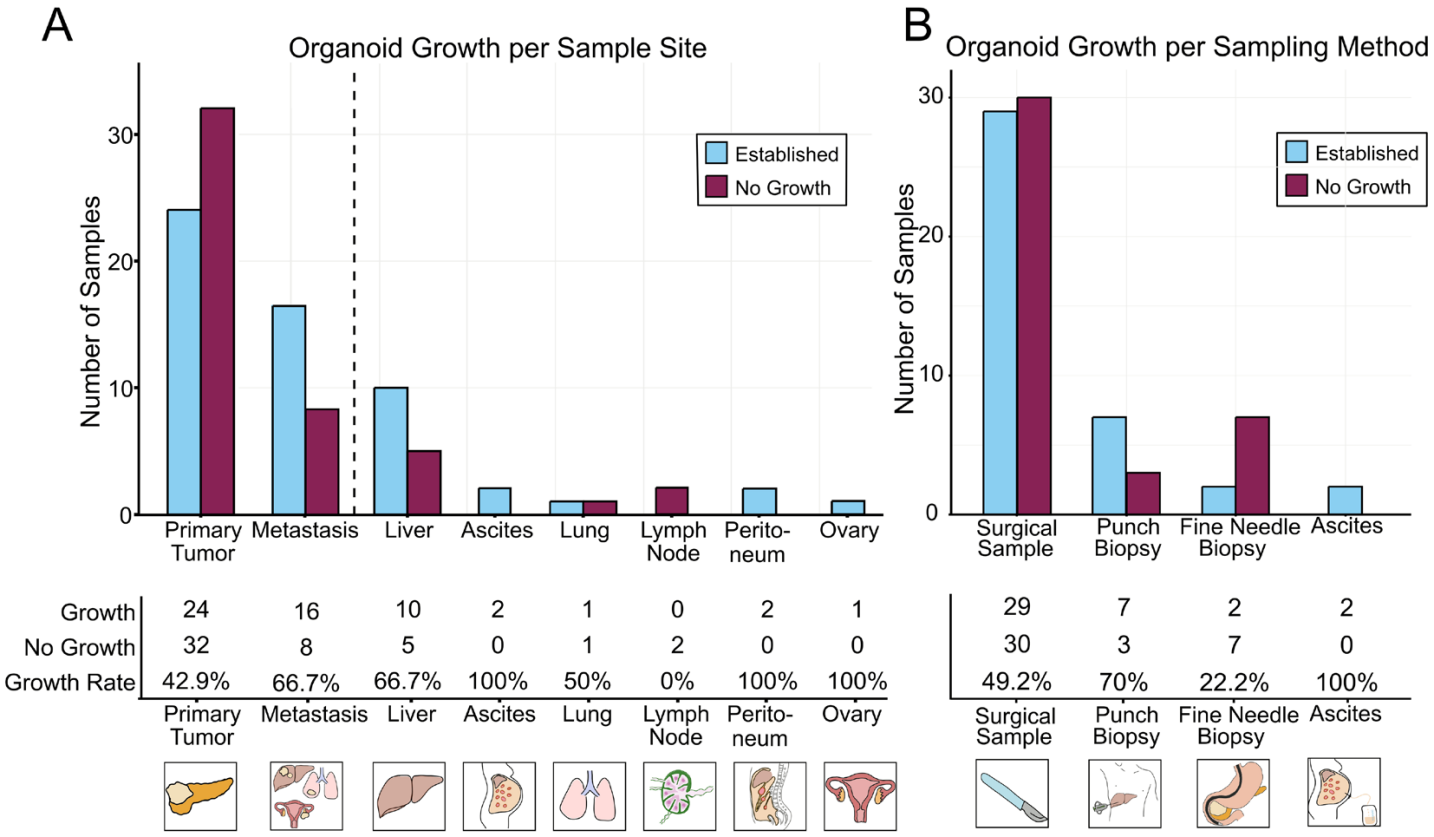
Organoid growth depends on sample site and sample type. (A) Organoids established from ascites and liver, peritoneal and ovarian metastases had higher establishment rates than organoid established from the primary tumor and lung metastases. No organoids could be established from lymph nodes. (B) Different sample types yielded varying establishing rates with ascites having the highest and FNBs the lowest growth rate.

Most samples were collected from primary tumors and liver metastases (Figure 3A). Overall, 42.9% (*n* = 24/56) of primary tumor samples and 66.7% (*n* = 16/24) of metastatic samples were successfully established. Establishment rates varied between the different metastatic sites. PDOs originating from peritoneal metastases, ascites and ovarian metastases were maintained in 100% of all cases. In contrast, we were not able to establish PDOs from lymph node metastases (Figure 3A). Moreover, among metastatic patients, samples derived from the primary tumor were observed to have significantly lower establishment rates than from the metastases (27.8% vs. 72.7%, OR = 4.67, *p* = 0.006, Table S2). Next, the effect of the sample type was assessed (Figure 3B). There was a significant difference in growth between FNBs and punch biopsies (*p* = 0.047).

As sample type was a potential confounder for the correlation between sample site and successful PDO growth, further confounding effects were assessed. Since the case numbers per sample type, however, were too small to perform a regression analysis, a direct evaluation of confounding effects was not possible. Instead, we performed a separate analysis of a subset of surgical samples only. Metastatic surgical samples had a higher establishment rate of 58.3% (7/12) compared to 46.8% (22/47) in primary tumors (Table S3). The univariate analysis yielded a trend towards better growth of metastatic samples but without statistical significance (OR = 1.59, *p* = 0.478).

#### 3.3.2 Uni‐ and Multivariate Analysis

We performed a univariate analysis to assess the impact of clinical and histopathological parameters on PDO growth (Table 2, Tables S4–S7 and Figures S1–S4). The most significant clinical parameters influencing PDO growth were CA 19–9 values above 500 U/mL (*p* = 0.045, OR = 4.00), diabetes at the time of sample acquisition (*p* = 0.043, OR = 0.18), BMI (*p* = 0.066, OR = 0.86), tumor site (*p* = 0.051, OR = 3.50) and age below 65 years (*p* = 0.087, OR = 2.67). For the multivariate analysis, age, CA 19‐9 levels and tumor site were assessed. Tumor site and CA 19‐9 levels above 500 U/L were found to have a significant impact on PDO growth in the multivariate analysis (Table 2 and Figures S3 and S4). Tumor cellularity, Ki‐67 expression, the percentage of necrotic tumor cells and TNM stage did not impact PDO growth (Tables S6 and S7 and Figures S1–S3). High GATA6 expression in the FFPE tissue, a marker for the classical PDAC subtype [41], was correlated with more successful PDO growth (*p* = 0.047, OR = 8.17, Tables S6 and S7, Figures S1–S3).

**TABLE 2:** Impact of age, CA19‐9 levels and tumor site on PDO growth.

Variable		Univariate analysis		Multivariate analysis	
		OR (CI)	*p*	OR (CI)	*p*
Age	> 65 years < 65 years	2.58 (0.83–8.23)	0.112	5.10 (0.98–37.3)	0.073
CA 19‐9	> 500 U/mL < 500 U/mL	4.00 (1.09–17.27)	0.045	5.71 (1.25–35.3)	0.037
Tumor site	Metastasis Primary tumor	3.50 (1.06–13.89)	0.051	8.13 (1.47–74.2)	0.031

### 3.4 Predictive and Prognostic Potential of PDO Growth

**FIGURE 4:**
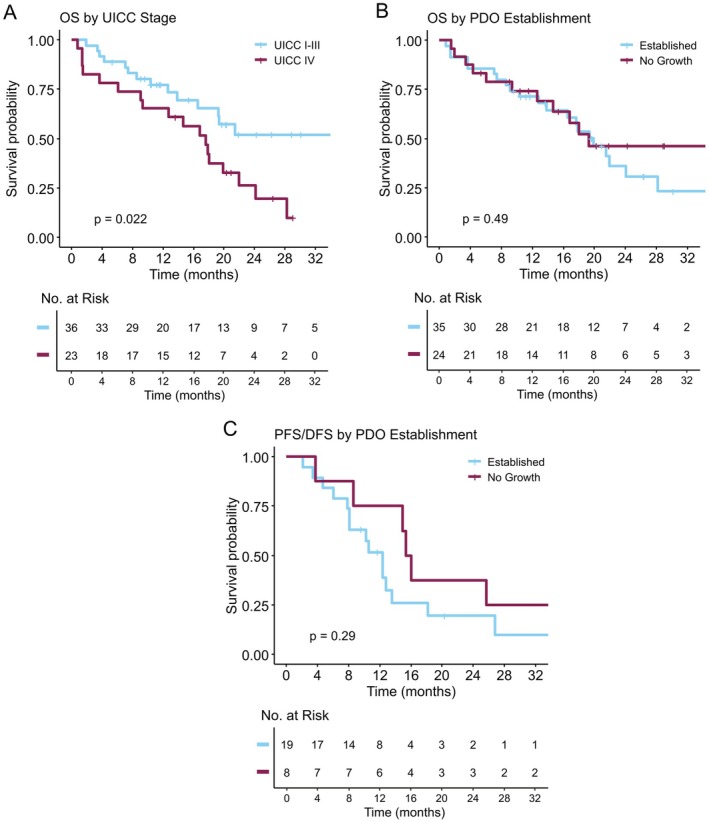
Kaplan–Meier curves for disease stage and organoid growth. (A) Shorter survival times were observed in patients with stage IV tumors in comparison to lower disease stages. (B) Sustained PDO growth had no prognostic potential for patients' survival. (C) We observed a trend between DFS/PFS and PDO growth, but this was not statistically significant.

Ultimately, we aimed to assess the predictive and prognostic potential of PDO establishment for patients' survival. We were able to include 59 patients for survival analyses. Two patients were excluded from survival analyses as one patient deceased intraoperatively and a second patient deceased from postoperative complications. Median OS (mOS) across all stages was 19.3 months in our patient cohort. For patients with resectable disease, the median survival was not reached within the follow‐up time. Patients with UICC Stage IV had a mOS of 17.6 months (*p* = 0.022, see Figure 4A). Factors influencing patient survival are summarized in Table S8. DFS was known for 20 patients with resectable disease (Figure S5C). PFS was available for 7 patients with metastatic disease (Figure S5D). We observed a trend towards longer DFS/PFS in patients without PDO growth, but this was not statistically significant (15.7 vs. 12.3 months, *p* = 0.29, Figure 4C). We did not observe a significant correlation between establishment and OS (19.9 months vs. 19.2 months, *p* = 0.58, Figure 4B).

## 4 Discussion

In this prospective observational study, we investigated the feasibility for clinical integration of PDO technology, predictive factors for PDO growth as well as the prognostic implication of PDO growth on DFS/PFS and OS. Previous research mainly focused on patho‐biological, but only a few on clinical variables. Thus, we aimed at addressing this knowledge gap by investigating the influence of clinical parameters on PDO growth.

The patient cohort comprising 62 PDAC patients was most representative for the resectable stage. For patients with resectable disease, mOS was similar to previously reported outcomes (2 year survival rate 52 vs. 40%–60%) [42, 43, 44]. In patients with metastatic PDAC, the mOS exceeded what is typically observed in real‐world settings (17.6 vs. 5.4 months) [45]. Therefore, a selection bias might be present in this particular patient cohort. Further sub‐analyses revealed metastatic patients to be younger than average PDAC patients (69.5 vs. 72–76 years) [46].

Translation of PDO technology into clinical practice is mostly limited by rates and time to PDO establishment. Previous studies on PDAC PDOs reported time trajectories from 3 to 16 weeks until pharmacotyping was realized [18, 28]. Our study has shown similar results with PDO establishment being feasible in a median of 4 weeks. This time frame works well for resected patients as adjuvant therapy is usually administered 6 to 12 weeks after resection [47]. Metastatic patients, on the other hand, are not surgically resected and therapy is initiated within a couple of days after histological verification. Therefore, PDO‐based therapies can easily be applied on resected PDAC patients while the time needed for PDO establishment appears too long for palliative treatment initiation. For immediate in vitro therapy testing, other technologies such as rapid pharmacotyping assays might be more beneficial. These assays potentially provide pharmacotyping data within 1–2 weeks after sampling, making treatment prediction feasible in the metastatic setting [17, 48, 49].

While these results are promising for future clinical applications, the overall PDO establishment rates remain an important bottleneck. In a recently published study, the pooled PDO establishment rate across several studies was 61.5% [50], which is in high accordance with the results of our study (establishment rate of 58%). The current establishment rates are insufficient for clinical application and require optimization for effective translation. We found a significant correlation between higher CA 19‐9 levels and PDO growth. Furthermore, a trend towards higher establishment rates was observed for metastatic samples. Similar results have previously been reported in PDX models of triple‐negative breast cancer [30] and PDO models derived from non‐small cell lung cancer [51]. Metastatic tumor cells are capable of undergoing both epithelial‐to‐mesenchymal and mesenchymal‐to‐epithelial transitions. This enables cells to adapt to a variety of tissue and culture conditions [52, 53, 54]. Furthermore, tumor stroma content is lower in PDAC metastases than in the original tumor [55, 56]. Metastatic samples might therefore be less prone to fibroblast overgrowth and have higher tumor cell content [18, 37]. CA 19‐9 is the standard tumor marker for PDAC indicating the tumor's activity and tumor burden. Higher CA 19‐9 is correlated with worse patient outcomes and higher tumor burden [57], indicating that more aggressive tumors tend to grow better in cell culture [58].

When considering immunohistopathological parameters, previous studies reported high tumor cell content [13, 18, 25, 27] and cell viability [25] to be positively correlated with PDO growth. This was not supported by our findings. High GATA6 expression was significantly correlated with successful PDO growth in our study. Strong GATA6 expression is a marker for the classical subtype of PDAC [41], which is associated with a better prognosis and better response towards mFOLFIRINOX [59]. It has previously been shown that the establishment rate of the classical subtype is higher [60]. This could be due to media conditions, as adding TGF‐beta leads to a shift towards more basal‐like organoids. Furthermore, the tumor microenvironment is missing in purely epithelial PDO models, which has been described to favor the classical subtype [60].

Moreover, PDO growth has been reported to be predictive for DFS/PFS and prognostic for OS in PDAC [32]. In our study, we saw a trend towards longer DFS in patients without PDO growth. This can be explained by the fact that more aggressive tumors relapse at a higher rate and respond less to adjuvant chemotherapy. To assess the correlation of PDO growth with PFS in metastatic patients, the sub‐cohort of only 7 metastatic patients was too small. OS did not correlate with PDO growth in our study, in contrast to a report by Boilève et al. [27], who described a correlation of OS with successful PDO growth.

There are several limitations to our study. First, while the follow‐up rate with documented OS in our study was high (61 out of 62 patients), the information on patients' treatments was unknown in 56% of patients. Second, we included a heterogeneous patient cohort of resected and metastasized tumor stages as well as samples from different sample sites and derived by various sample types. Furthermore, several subgroup analyses, such as assessing the influence of patients' pretreatment, diabetes, or BMI, were limited due to the low number of patients per group. For histopathological staining, a different region of the tumor was preserved as an FFPE block than the region used to generate the PDO models. Due to intratumoral heterogeneity, this could have resulted in differing levels of histopathological markers between the two samples.

Overall, we showed that the time trajectory for PDO establishment is clinically feasible. However, PDO establishment rates of 60% limit a broader clinical implementation, with the highest rates being achieved from metastatic tissues. One way to further increase establishment rates might be achieved through protocol adaptations. Driehuis et al. [13] underlined the importance of live‐monitored digestion of tissue samples to prevent over‐digestion. Furthermore, current PDO culture media compositions favor classical‐like PDOs, with the establishment of basal‐like subtypes being under‐represented [60], which was supported by our findings. Adaptation of culture media might therefore be central to improving the overall yield of PDO growth. Nonetheless, establishment rates remain a significant limitation of PDO technology. As PDOs are resource‐intensive, expensive, and currently only feasible in translational research centers with highly trained lab personnel [61], future broad clinical application will require considerable efforts. Though, PDO pharmacotyping seems feasible and was also previously shown to correlate with the response of patients presented in this study [34]. Nonetheless, PDO technology has great potential to improve our understanding of PDAC biology. Existing biobank repositories are of significant value for future translational research, as they provide a group of models that can be matched to clinically relevant subgroups (e.g., specific KRAS mutations). This will be of particular interest, for example, when addressing the development of resistance to KRAS inhibitors. Moreover, PDO biobanks can be used for high‐throughput screening approaches to identify new targeted treatment options [48, 49].

## Author Contributions

Conception and design: C.C.M.N., K.W., U.P. Administrative support: C.C.M.N., U.P., U.K., S.S. Provision of study materials or patients: M.F., M.L., C.J., M.B. Collection and assembly of data: K.W., F.S., F.D., A.K., M.P.D., M.J., C.C.M.N. Data analysis and interpretation: C.C.M.N., K.W., F.S., M.P.D., U.P., U.K. Manuscript writing: C.C.M.N., K.W., F.S., F.D., A.K., M.P.D., M.J., G.H., L.V., M.F., M.L., C.J., M.B., G.D., R.S., S.S., U.K., U.P. Final approval of manuscript: C.C.M.N., K.W., F.S., F.D., A.K., M.P.D., M.J., G.H., L.V., M.F., M.L., C.J., M.B., G.D., R.S., S.S., U.K., U.P.

## Funding

This research was funded by the Berliner Krebsgesellschaft (Grant PEFF202104) and by the “Hans Beger Stiftung zur Erforschung und Bekämpfung von Bauchspeicheldrüsenkrebs”. Prof. Dr. Pelzer was a participant of the BIH‐Charité Clinical Fellow, Dr. Neumann of the BIH‐Charité Junior Clinician Scientist program and K. Wansch a BIH MD Doctoral scholar funded by the Charité‐Universitätsmedizin Berlin and the Berlin Institute of Health. Dr. Dragomir was supported by the Berlin Institute of Health (Clinician Scientist Program) and by the DKTK Berlin (Young Investigator Grant 2022). Dr. Dragomir, Dr. Hilfenhaus, Dr. Vecchione and PD Dr. Felsenstein were supported by the Berlin Institute of Health (Clinician Scientist Program).

## Ethics Statement

The authors are accountable for all aspects of the work in ensuring that questions related to the accuracy or integrity of any part of the work are appropriately investigated and resolved. The study was conducted in accordance with the Declaration of Helsinki (as revised in 2013). The study was approved by the ethics board of the Charité‐Universitätsmedizin Berlin (No. EA1/157/21) and informed consent was obtained from all individual participants.

## Consent

The authors have nothing to report.

## Conflicts of Interest

The authors declare no conflicts of interest.

## Supporting information


**Data S1:** cam471490‐sup‐0001‐Supplementary File 1.docx.

## Data Availability

All data generated or analyzed during this study are included in this published article and its Supporting Information files.
