# Supplementary material for: Identifying Factors of Organoid Establishment in Pancreatic Cancer: A Prospective Observational Study
Source: Cancer Med. 2025 Dec 30;15(1):e71490. doi: 10.1002/cam4.71490 (PMC12753581; doi:10.1002/cam4.71490)
Supplement: Supplementary file 1 — Data S1: cam471490‐sup‐0001‐Supplementary File 1.docx. [file CAM4-15-e71490-s001.docx]

**Supplementary File 1**

**Supplementary Table 1: Clinical parameters of all patients enrolled in the study**

|  | **Overall (N=75)** |
| --- | --- |
| **Age** |  |
| Median (Min – Max) | 69.0 (41.0 - 91.0) |
| **Sex** |  |
| Female | 43 (57.3%) |
| Male | 32 (42.7%) |
| **Stage** |  |
| Resectable | 48 (64.0%) |
| Metastasized | 26 (34.7%) |
| Missing | 1 (1.3%) |
| **Treatment** |  |
| Therapy-naive | 61 (81.3%) |
| Neoadjuvant treatment | 8 (10.7%) |
| Missing | 6 (8.0%) |

**Supplementary Table 2: Growth rate of samples from Patients with Resectable and Patients with Metastastic Disease**

|  | **Resectable Disease** | | **Metastatic Disease** | |
| --- | --- | --- | --- | --- |
|  | **Primary Tumor,**  **N=38 (%)** | **Lymph Node, N=2 (%)** | **Primary Tumor, N=18 (%)** | **Metastasis,**  **N=22 (%)** |
| **Established** | 19 (50.0) | 0 (0) | 5 (27.8) | 16 (72.7) |
| **No Growth** | 19 (50.0) | 2 (100) | 13 (72.2) | 6 (27.3) |

**Supplementary Table 3: Growth rate of Surgical Samples**

|  | **Overall,**  **N=59 (%)** | **Primary Tumor,**  **N=47 (%)** | **Metastasis, N=12 (%)** |
| --- | --- | --- | --- |
| **Established** | 29 (49.2) | 22 (46.8) | 7 (58.3) |
| **No Growth** | 30 (50.8) | 25 (53.2) | 5 (41.7) |

**Supplementary Table 4: Summary of laboratory parameters for PDAC patients**

|  | **Overall (N=62)** | **Established (N=36)** | **No Growth (N=26)** |
| --- | --- | --- | --- |
| **Haemoglobin (g/dl)** |  |  |  |
| Median (Min - Max) | 12.6 (6.90 - 17.2) | 12.8 (6.90 - 16.0) | 12.0 (9.10 - 17.2) |
| **Platelets (/nl)** |  |  |  |
| Median (Min - Max) | 255 (83.0 - 897) | 252 (84.0 - 714) | 257 (83.0 - 897) |
| **Leukocytes (/nl)** |  |  |  |
| Median (Min - Max) | 7.57 (3.12 - 19.2) | 7.46 (3.96 - 19.0) | 8.17 (3.12 - 19.2) |
| **Lymphocytes (/nl)** |  |  |  |
| Median (Min - Max) | 1.33 (0.460 - 2.74) | 1.44 (0.380 - 2.65) | 1.40 (0.380 - 2.74) |
| Missing | 13 (50.0%) | 16 (44.4%) | 29 (46.8%) |
| **Monocytes (/nl)** |  |  |  |
| Median (Min - Max) | 0.630 (0.340 - 1.70) | 0.61 (0.34 - 0.90) | 0.685 (0.46 - 1.70) |
| Missing | 29 (46.8%) | 15 (41.7%) | 14 (53.8%) |
| **Neutrophils (/nl)** |  |  |  |
| Median (Min - Max) | 5.14 (1.61 - 16.9) | 5.14 (1.61 - 16.9) | 4.92 (3.87 - 14.4) |
| Missing | 29 (46.8%) | 15 (41.7%) | 14 (53.8%) |
| **NLR** |  |  |  |
| Median (Min - Max) | 3.75 (1.08 - 22.9) | 2.93 (1.08 - 22.9) | 4.62 (1.41 - 8.96) |
| Missing | 30 (48.4%) | 16 (44.4%) | 14 (53.8%) |
| **CAR** |  |  |  |
| Median (Min - Max) | 0.0833 (0.00209 - 2.65) | 0.075 (0.002 - 2.65) | 0.105 (0.01 - 1.97) |
| Missing | 25 (40.3%) | 11 (30.6%) | 14 (53.8%) |
| **LMR** |  |  |  |
| Median (Min - Max) | 2.23 (0.543 - 6.63) | 2.31 (0.54 - 6.63) | 1.69 (1.00 - 4.03) |
| Missing | 30 (48.4%) | 16 (44.4%) | 14 (53.8%) |
| **PLR** |  |  |  |
| Median (Min - Max) | 193 (63.7 - 657) | 203 (83.7 - 389) | 189 (63.7 - 657) |
| Missing | 29 (46.8%) | 16 (44.4%) | 13 (50.0%) |
| **IBI** |  |  |  |
| Median (Min - Max) | 73.6 (3.68 - 503) | 80.7 (3.68 - 503) | 46.7 (9.42 - 380) |
| Missing | 30 (48.4%) | 16 (44.4%) | 14 (53.8%) |
| **CRP (mg/l)** |  |  |  |
| Median (Min - Max) | 3.00 (0.100 - 74.7) | 1.80 (0.100 - 64.3) | 4.10 (0.600 - 74.7) |
| Missing | 3 (4.8%) | 0 (0%) | 3 (11.5%) |
| **LDH (U/l)** |  |  |  |
| Median (Min - Max) | 231 (132 - 446) | 234 (132 - 446) | 228 (153 - 429) |
| Missing | 24 (38.7%) | 11 (30.6%) | 13 (50.0%) |
| **CA 19-9 (U/ml)** |  |  |  |
| Median (Min - Max) | 388 (2.00 - 30300) | 623 (2.00 - 30300) | 143 (7.10 - 1960) |
| Missing | 18 (29.0%) | 8 (22.2%) | 10 (38.5%) |
| **CEA (µg/l)** |  |  |  |
| Median (Min - Max) | 5.19 (0.700 - 698) | 4.33 (0.700 - 124) | 5.20 (0.930 - 698) |
| Missing | 21 (33.9%) | 11 (30.6%) | 10 (38.5%) |
| **Albumin (g/l)** |  |  |  |
| Median (Min - Max) | 42.3 (23.0 - 49.1) | 41.0 (23.0 - 49.1) | 42.9 (35.3 - 45.9) |
| Missing | 25 (40.3%) | 11 (30.6%) | 14 (53.8%) |
| **Bilirubin (mg/dl)** |  |  |  |
| Median (Min - Max) | 0.700 (0.200 - 16.9) | 0.71 (0.20 - 14.1) | 0.63 (0.20 - 16.9) |
| Missing | 1 (1.6%) | 1 (2.8%) | 0 (0%) |
| **GGT (U/l)** |  |  |  |
| Median (Min - Max) | 74.0 (9.00 - 2370) | 87.0 (9.00 - 2370) | 60.5 (18.0 - 1310) |
| Missing | 1 (1.6%) | 1 (2.8%) | 0 (0%) |
| **INR** |  |  |  |
| Median (Min - Max) | 1.01 (0.800 - 3.96) | 1.02 (0.80 - 3.96) | 0.99 (0.80 - 1.21) |
| **aPTT (s)** |  |  |  |
| Median (Min - Max) | 28.0 (22.0 - 45.2) | 29.3 (22.0 - 45.2) | 27.6 (23.6 - 39.9) |
| Missing | 1 (1.6%) | 1 (2.8%) | 0 (0%) |

**Supplementary Table 5: TNM stage and organoid growth**

|  | **Overall (N=38)** | **Established (N=19)** | **No Growth (N=19)** |
| --- | --- | --- | --- |
| **T-Stage** |  |  |  |
| T1 | 4 (10.5%) | 3 (15.8%) | 1 (5.3%) |
| T2 | 21 (55.3%) | 12 (63.2%) | 9 (47.4%) |
| T3 | 12 (31.6%) | 4 (21.1%) | 8 (42.1%) |
| T4 | 1 (2.6%) | 0 (0%) | 1 (5.3%) |
| **N-Stage** |  |  |  |
| N0 | 12 (31.6%) | 5 (26.3%) | 7 (36.8%) |
| N1 | 16 (42.1%) | 9 (47.4%) | 7 (36.8%) |
| Nx | 10 (26.3%) | 5 (26.3%) | 5 (26.3%) |
| **Lymphovascular invasion** |  |  |  |
| L0 | 21 (55.3%) | 11 (57.9%) | 10 (52.6%) |
| L1 | 16 (42.1%) | 8 (42.1%) | 8 (42.1%) |
| Lx | 1 (2.6%) | 0 (0%) | 1 (5.3%) |
| **Perineural invasion** |  |  |  |
| Pn0 | 5 (13.2%) | 5 (26.3%) | 0 (0%) |
| Pn1 | 32 (84.2%) | 14 (73.7%) | 18 (94.7%) |
| Pnx | 1 (2.6%) | 0 (0%) | 1 (5.3%) |
| **Vascular invasion** |  |  |  |
| V0 | 34 (89.5%) | 18 (94.7%) | 16 (84.2%) |
| V1 | 4 (10.5%) | 1 (5.3%) | 3 (15.8%) |
| **Grading** |  |  |  |
| G1 | 20 (52.6%) | 11 (57.9%) | 9 (47.4%) |
| G2 | 8 (21.1%) | 5 (26.3%) | 3 (15.8%) |
| G3 | 9 (23.7%) | 3 (15.8%) | 6 (31.6%) |
| Gx | 1 (2.6%) | 0 (0%) | 1 (5.3%) |

**Supplementary Table 6: Histological and immunohistochemical characteristics in the FFPE tissue of a subset of 20 matched patients**

|  | **Overall (N=20)** | **Established (N=10)** | **No Growth (N=10)** |
| --- | --- | --- | --- |
| **Ki-67** |  |  |  |
| Median (Min - Max) | 0.40 (0.15 - 0.80) | 0.28 (0.15 - 0.80) | 0.40 (0.15 - 0.70) |
| **Tumor Cellularity** |  |  |  |
| Median (Min - Max) | 0.35 (0.10 - 0.60) | 0.35 (0.10 - 0.60) | 0.40 (0.10 - 0.60) |
| Missing | 1 (5.0%) | 1 (10.0%) | 0 (0.0%) |
| **Necrosis** |  |  |  |
| Median (Min - Max) | 0.02 (0 - 0.85) | 0 (0 - 0.07) | 0.04 (0 - 0.85) |
| Missing | 1 (5.0%) | 1 (10.0%) | 0 (0.0%) |
| **GATA6** |  |  |  |
| Median (Min - Max) | 3.00 (1.00 - 4.00) | 3.00 (2.00 - 4.00) | 2.00 (1.00 - 4.00) |
| Missing | 1 (5.0%) | 1 (10.0%) | 0 (0.0%) |
| **GATA6 Grading** |  |  |  |
| High expression | 10 (16.1%) | 7 (19.4%) | 3 (11.5%) |
| Low expression | 9 (14.5%) | 2 (5.6%) | 7 (26.9%) |
| Missing | 1 (5.0%) | 1 (10.0%) | 0 (0.0%) |

**Supplementary Table 7: Univariate Analysis of factors influencing PDO growth**

| **Variable** | | **Odds Ratio (CI)** | | **P-Value** | |
| --- | --- | --- | --- | --- | --- |
| **Pretreatment** | Untreated  Treated | 0.91 (0.21 – 3.57) | 0.892 | |  |
| **Sex** | Male  Female | 0.45 (0.15 – 1.29) | 0.139 | |  |
| **Age** |  | 0.97 (0.92 – 1.02) | 0.238 | |  |
| **Age (Cut Off)** | <65 years  >65 years | 2.67 (0.9 – 8.73) | 0.087 | |  |
| **Stage** | Metastasized  Local | 2.43 (0.84 – 7.54) | 0.109 | |  |
| **ECOG** | I-II  III-IV | 0.27 (0.01 – 1.91) | 0.254 | |  |
| **Tumor Site** | Metastasis  Primary | 3.50 (1.06 – 13.89) | 0.051 | |  |
| **Diabetes** | Yes  No | 0.18 (0.03 – 0.89) | 0.043 | |  |
| **BMI (kg/m²)** |  | 0.86 (0.72 – 1.00) | 0.066 | |  |
| **Ki-67** |  | 0.46 (0.003 – 52.09) | 0.735 | |  |
| **Tumor Cellularity** |  | 1.09 (0.003 – 467.4) | 0.975 | |  |
| **Albumin (g/l)** |  | 0.96 (0.80 – 1.14) | 0.626 | |  |
| **Albumin (Cut Off)** | >30 g/l  <30 g/l | NA | | NA | |
| **Bilirubin (mg/dl)** |  | 1.04 (0.89 – 1.23) | 0.646 | |  |
| **Bilirubin (Cut Off)** | >1.1 mg/dl  <1.1 mg/dl | 0.83 (0.39 – 2.42) | | 0.737 | |
| **GGT (U/l)** |  | 1.00 (1.00 – 1.00) | 0.873 | |  |
| **GGT (Cut Off)** | >40 U/l  <40 U/l | 1.81 (0.60 – 5.50) | | 0.994 | |
| **Haemoglobin (g/dl)** |  | 1.02 (0.79 – 1.31) | 0.894 | |  |
| **Haemoglobin (Cut Off)** | >10 g/dl  <10 g/dl | 1.43 (0.25 – 8.36) | | 0.675 | |
| **Leukocytes (/nl)** |  | 0.90 (0.71 – 1.13) | 0.224 | |  |
| **Lymphocytes (/nl)** |  | 1.19 (0.31 – 4.84) | | 0.794 | |
| **Lymphocyte (Cut Off)** | >1.35 /nl  <1.35 /nl | 2.17 (0.53 – 9.42) | 0.288 | |  |
| **Platelets (/nl)** |  | 1.00 (0.99 – 1.00) | 0.588 | |  |
| **Platelets (Cut Off)** | >235 /nl  <235 /nl | 1.80 (0.62 – 5.40) | 0.285 | |  |
| **Monocytes (/nl)** |  | 0.21 (0.00 – 20.39) | 0.509 | |  |
| **Monocytes (Cut Off)** | >0.6 /nl  <0.6 /nl | 0.50 (0.09 – 2.31) | 0.391 | |  |
| **Neutrophils (/nl)** |  | 0.87 (0.57 – 1.30) | 0.493 | |  |
| **Neutrophil (Cut Off)** | >5 /nl  <5 /nl | 1.20 (0.27 – 5.44) | 0.810 | |  |
| **CRP (mg/l)** |  | 0.99 (0.96 – 1.03) | 0.713 | |  |
| **CRP (Cut Off)** | >5 mg/l  <5 mg/l | 0.83 (0.27 – 2.56) | 0.735 | |  |
| **INR** |  | 6.03 (0.04 – 1111.29) | 0.482 | |  |
| **aPTT (s)** |  | 1.05 (0.89 – 1.24) | 0.551 | |  |
| **CA 19-9 (U/ml)** |  | 1.00 (1.00 – 1.00) | 0.132 | |  |
| **CA 19-9 (Cut Off)** | >500 U/ml  <500 U/ml | 4,00 (1.09 – 17.27) | 0.045 | |  |
| **CEA (µg/l)** |  | 0.99 (0.97 – 1.00) | 0.265 | |  |
| **CEA (Cut Off)** | >7.2 µg/l  <7.2 µg/l | 0.94 (0.26 – 3.55) | | 0.923 | |
| **LDH (U/l)** |  | 1.00 (0.99 – 1.01) | 0.503 | |  |
| **LDH (Cut Off)** | >250 U/l | 0.78 (0.20 – 3.07) | | 0.716 | |
| **NLR** |  | 0.98 (0.80 – 1.23) | 0.867 | |  |
| **NLR (Cut Off)** | >4.0  <4.0 | 0.38 (0.08 – 1.75) | 0.221 | |  |
| **CAR** |  | 0.39 (0.04 – 2.22) | 0.305 | |  |
| **CAR (Cut Off)** | >0.4  <0.4 | 0.75 (0.11 – 6.39) | 0.772 | |  |
| **LMR** |  | 1.39 (0.72 – 3.36) | 0.385 | |  |
| **LMR (Cut Off)** | >1.6  <1.6 | 3.24 (0.57 – 20.40) | 0.185 | |  |
| **PLR** |  | 1.00 (0.99 – 1.00) | 0.723 | |  |
| **PLR (Cut Off)** | >180  <180 | 1.71 (0.39 – 7.68) | 0.471 | |  |
| **IBI** |  | 1.00 (1.00 – 1.01) | 0.311 | |  |
| **IBI (Cut Off)** | >30  <30 | 2.85 (0.50 – 18.13) | 0.238 | |  |
| **T-Stage** | T3-T4  T1-T2 | 0.67 (0.19 – 2.27) | 0.516 | |  |
| **N-Stage** | N1  N0 | 2.40 (0.56 – 11.15) | 0.246 | |  |
| **L-Stage** | L1  L0 | 0.91 (0.24 – 3.37) | 0.886 | |  |
| **Pn-Stage** | Pn1  Pn0 | NA | NA | |  |
| **V-Stage** | V1  V0 | 0.44 (0.02 – 5.07) | 0.524 | |  |
| **Necrosis** |  | NA | NA | |  |
| **GATA6** |  | 3.49 (1.12 – 16.95) | 0.060 | |  |
| **GATA6 Grading** | High expression  Low expression | 8.17 (1.17 – 83.38) | 0.047 | |  |

**Supplementary Table 8: Univariate Analysis of factors influencing patients’ OS**

| Variable | | Hazard Ratio (CI) | | P-Value | |
| --- | --- | --- | --- | --- | --- |
| **Pretreatment** | Untreated  Treated | 0.51 (0.24 – 1.11) | 0.091 | |  |
| **Sex** | Male  Female | 0.79 (0.38 – 1.68) | 0.544 | |  |
| **Age** |  | 1.02 (0.99 – 1.06) | 0.180 | |  |
| **Age (Cut Off)** | <65 years  >65 years | 0.73 (0.35 – 1.51) | 0.393 | |  |
| **Stage** | Metastasized  Local | 2.22 (1.10 – 4.47) | 0.026 | |  |
| **ECOG** | I-II  III-IV | 0.20 (0.08 – 0.51) | 0.001 | |  |
| **Diabetes** | Yes  No | 0.84 (0.28 – 2.54) | 0.763 | |  |
| **BMI (kg/m²)** |  | 1.01 (0.91 – 1.12) | 0.818 | |  |
| **Albumin (g/l)** |  | 1.03 (0.93 – 1.16) | 0.532 | |  |
| **Albumin (Cut Off)** | >30 g/l  <30 g/l | 0.25 (0.06 – 1.15) | | 0.075 | |
| **Bilirubin (mg/dl)** |  | 1.01 (0.90 – 1.13) | 0.835 | |  |
| **Bilirubin (Cut Off)** | >1.1 mg/dl  <1.1 mg/dl | 0.69 (0.31 – 1.54) | | 0. 360 | |
| **GGT (U/l)** |  | 1.00 (1.00 – 1.00) | 0.982 | |  |
| **GGT (Cut Off)** | >40 U/l  <40 U/l | 1.75 (0.78 – 23.94) | | 0.173 | |
| **Haemoglobin (g/dl)** |  | 1.00 (0.83 – 1.20) | 0.974 | |  |
| **Haemoglobin (Cut Off)** | >10 g/dl  <10 g/dl | 0.70 (0.24 – 1.99) | | 0.500 | |
| **Leukocytes (/nl)** |  | 1.03 (0.87 – 1.21) | 0.757 | |  |
| **Lymphocytes (/nl)** |  | 0.68 (0.29 – 1.62) | | 0.389 | |
| **Lymphocyte (Cut Off)** | >1.35 /nl  <1.35 /nl | 0.53 (0.20 – 1.39) | 0.195 | |  |
| **Platelets (/nl)** |  | 1.00 (1.00 – 1.00) | 0.664 | |  |
| **Platelets (Cut Off)** | >235 /nl  <235 /nl | 0.84 (0.42 – 1.68) | 0.615 | |  |
| **Monocytes (/nl)** |  | 11.88 (0.48 – 292.07) | 0.130 | |  |
| **Monocytes (Cut Off)** | >0.6 /nl  <0.6 /nl | 1.70 (0.63 – 4.63) | 0.411 | |  |
| **Neutrophils (/nl)** |  | 1.13 (0.87 – 1.45) | 0.360 | |  |
| **Neutrophil (Cut Off)** | >5 /nl  <5 /nl | 2.05 (0.75 – 5.58) | 0.161 | |  |
| **CRP (mg/l)** |  | 0.99 (0.96 – 1.02) | 0.662 | |  |
| **CRP (Cut Off)** | >5 mg/l  <5 mg/l | 1.35 (0.64 – 2.85) | 0.430 | |  |
| **INR** |  | 27.04 (0.87 – 836.90) | 0.060 | |  |
| **aPTT (s)** |  | 1.06 (0.94 – 1.18) | 0.350 | |  |
| **CA 19-9 (U/ml)** |  | 1.00 (1.00 – 1.00) | 0.162 | |  |
| **CA 19-9 (Cut Off)** | >500 U/ml  <500 U/ml | 1.13 (0.51 – 2.51) | 0.754 | |  |
| **CEA (µg/l)** |  | 1.00 (1.00 – 1.01) | 0.005 | |  |
| **CEA (Cut Off)** | >7.2 µg/l  <7.2 µg/l | 0.91 (0.39 – 2.15) | | 0.837 | |
| **LDH (U/l)** |  | 1.00 (1.00 – 1.01) | 0.279 | |  |
| **LDH (Cut Off)** | >250 U/l | 1.23 (0.53 – 2.86) | | 0.624 | |
| **NLR** |  | 1.04 (0.95 – 1.15) | 0.371 | |  |
| **NLR (Cut Off)** | >4.0  <4.0 | 1.42 (0.53 – 3.80) | 0.484 | |  |
| **CAR** |  | 0.27 (0.02 – 3.13) | 0.297 | |  |
| **CAR (Cut Off)** | >0.4  <0.4 | 0.40 (0.05 – 3.07) | 0.381 | |  |
| **LMR** |  | 0.74 (0.44 – 1.25) | 0.267 | |  |
| **LMR (Cut Off)** | >1.6  <1.6 | 0.65 (0.23 – 1.84) | 0.414 | |  |
| **PLR** |  | 1.00 (0.99 – 1.00) | 0.378 | |  |
| **PLR (Cut Off)** | >180  <180 | 0.67 (0.25 – 1.78) | 0.423 | |  |
| **IBI** |  | 1.00 (1.00 – 1.00) | 0.226 | |  |
| **IBI (Cut Off)** | >30  <30 | 0.81 (0.26 – 2.52) | 0.714 | |  |
| **T-Stage** | T3-T4  T1-T2 | 0.62 (0.22 – 1.73) | 0.363 | |  |
| **N-Stage** | N1  N0 | 1.54 (0.41 – 5-87) | 0.520 | |  |
| **L-Stage** | L1  L0 | 2.18 (0.74 – 6.37) | 0.156 | |  |
| **Pn-Stage** | Pn1  Pn0 | 0.47 (0.15 – 1.52) | 0.210 | |  |
| **V-Stage** | V1  V0 | 3.08 (0.68 – 14.05) | 0.146 | |  |

**
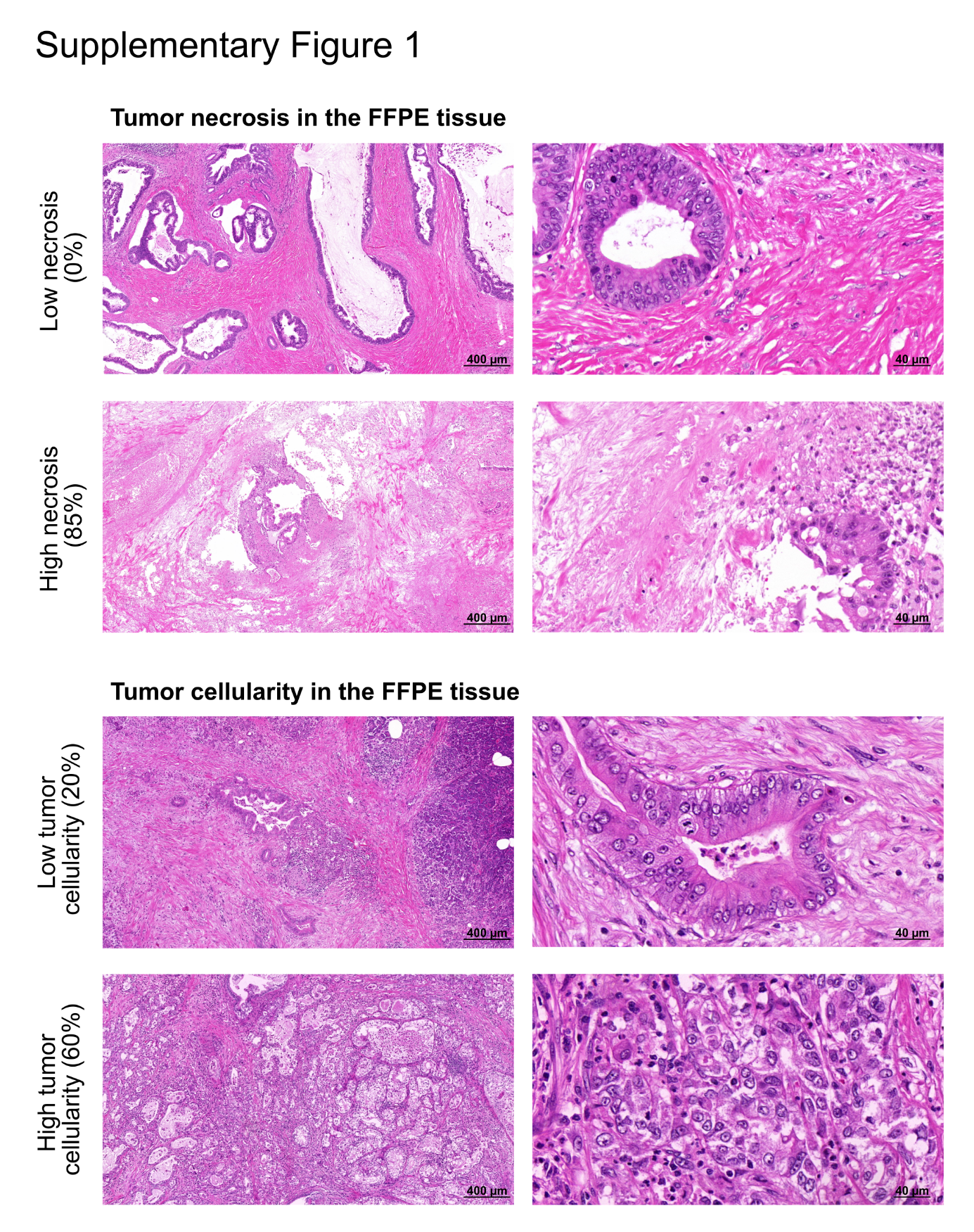
**

***Supplementary Figure 1: H&E-staining of FFPE tissue for evaluation of necrosis and tumor cellularity***

***
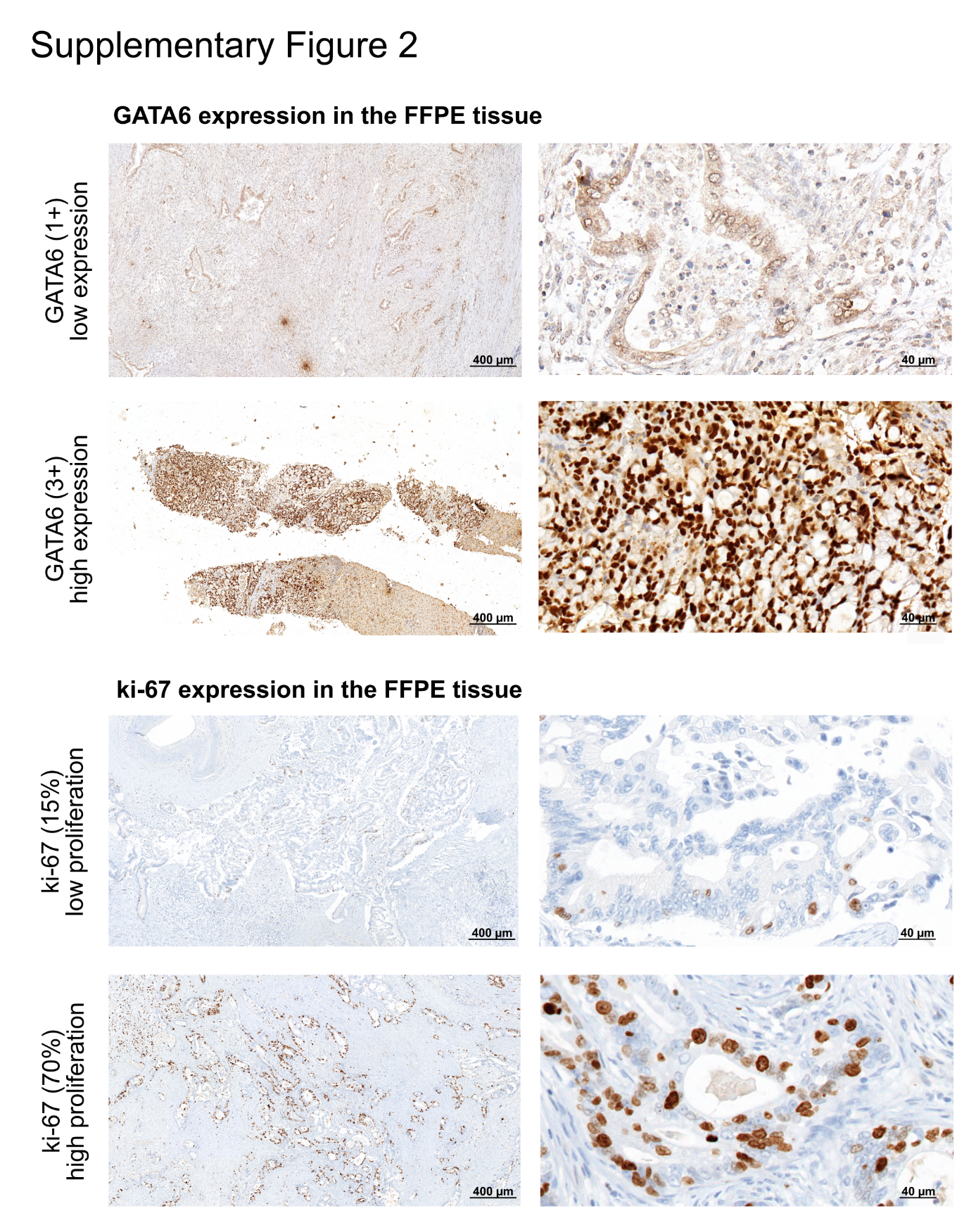
***

***Supplementary Figure 2: Immunohistochemical staining of GATA6 and Ki-67 for subtype classification and evaluation of proliferation in the FFPE tissue***

*
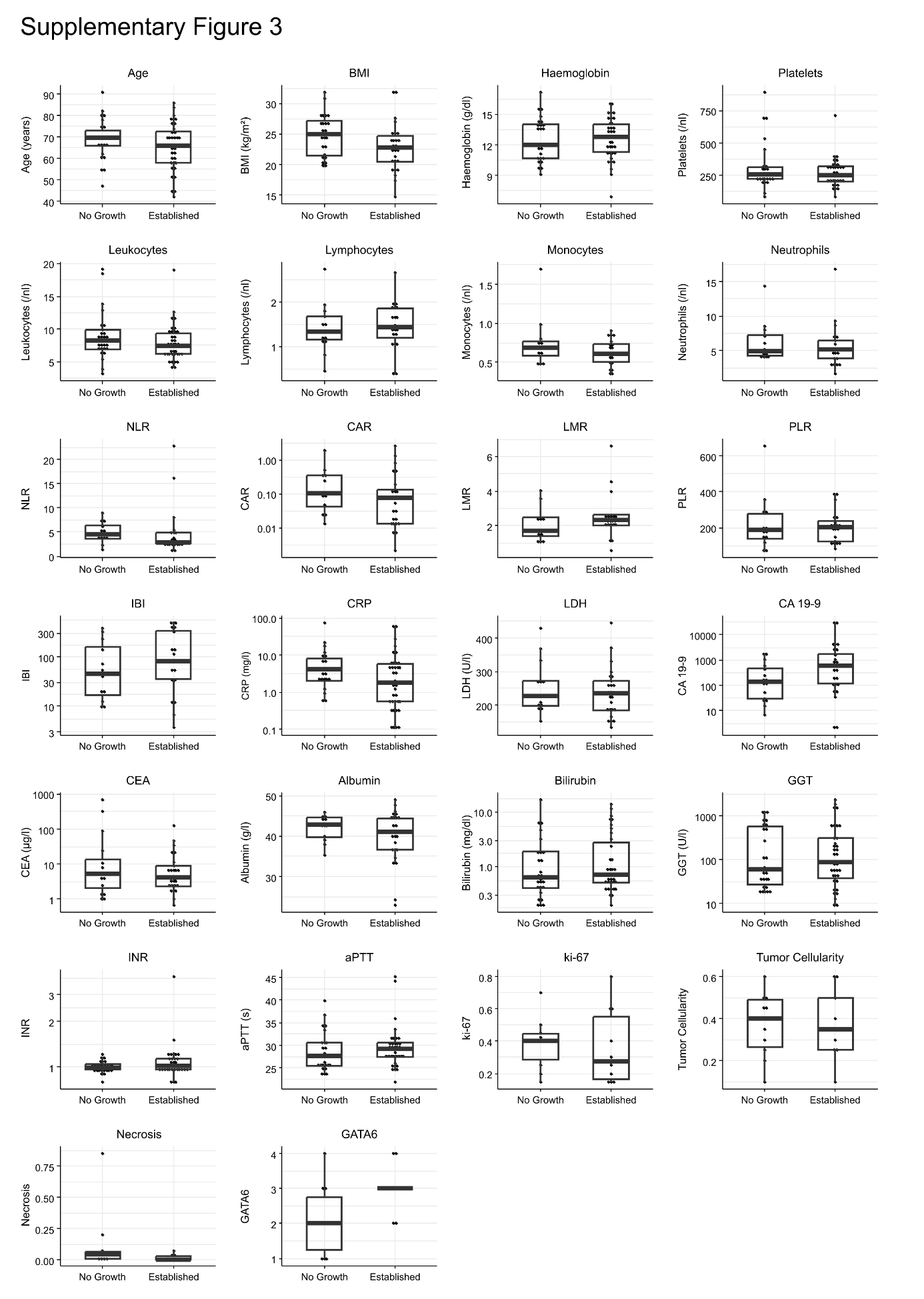
*

***Supplementary Figure 3: Boxplots of clinical and histopathological variables for patients with and without PDO growth***

***
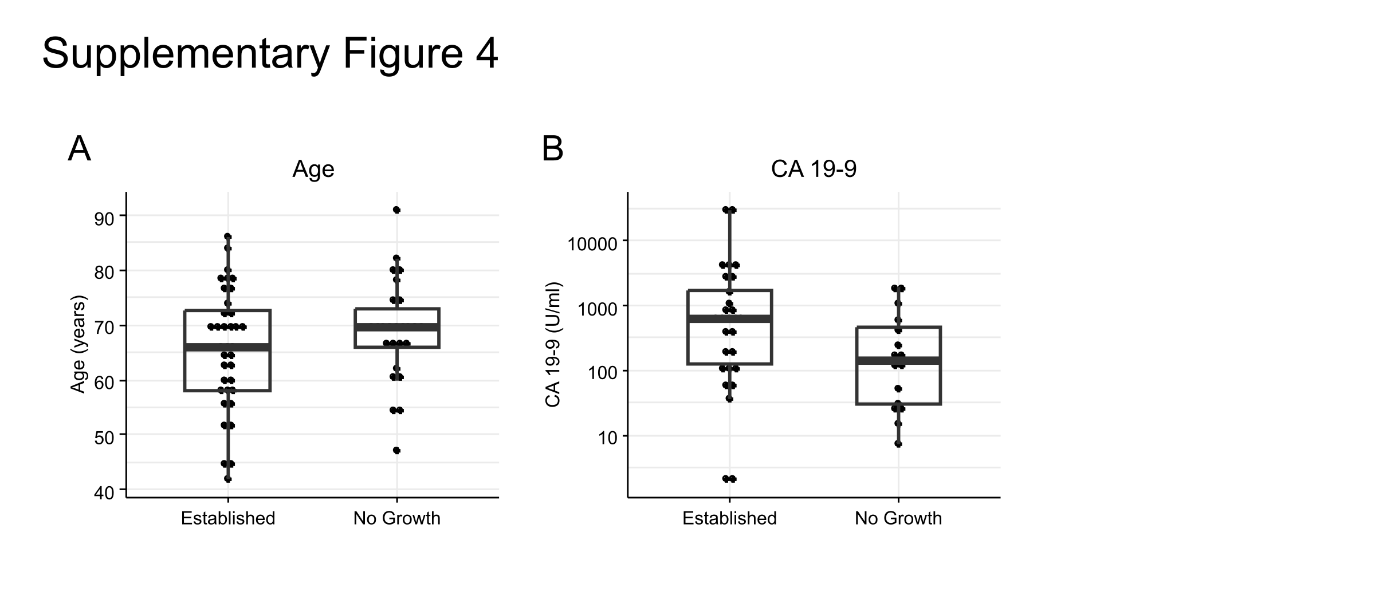
***

***Supplementary Figure 4: Boxplots of Age and CA 19-9 levels in patients with and without PDO growth***

***
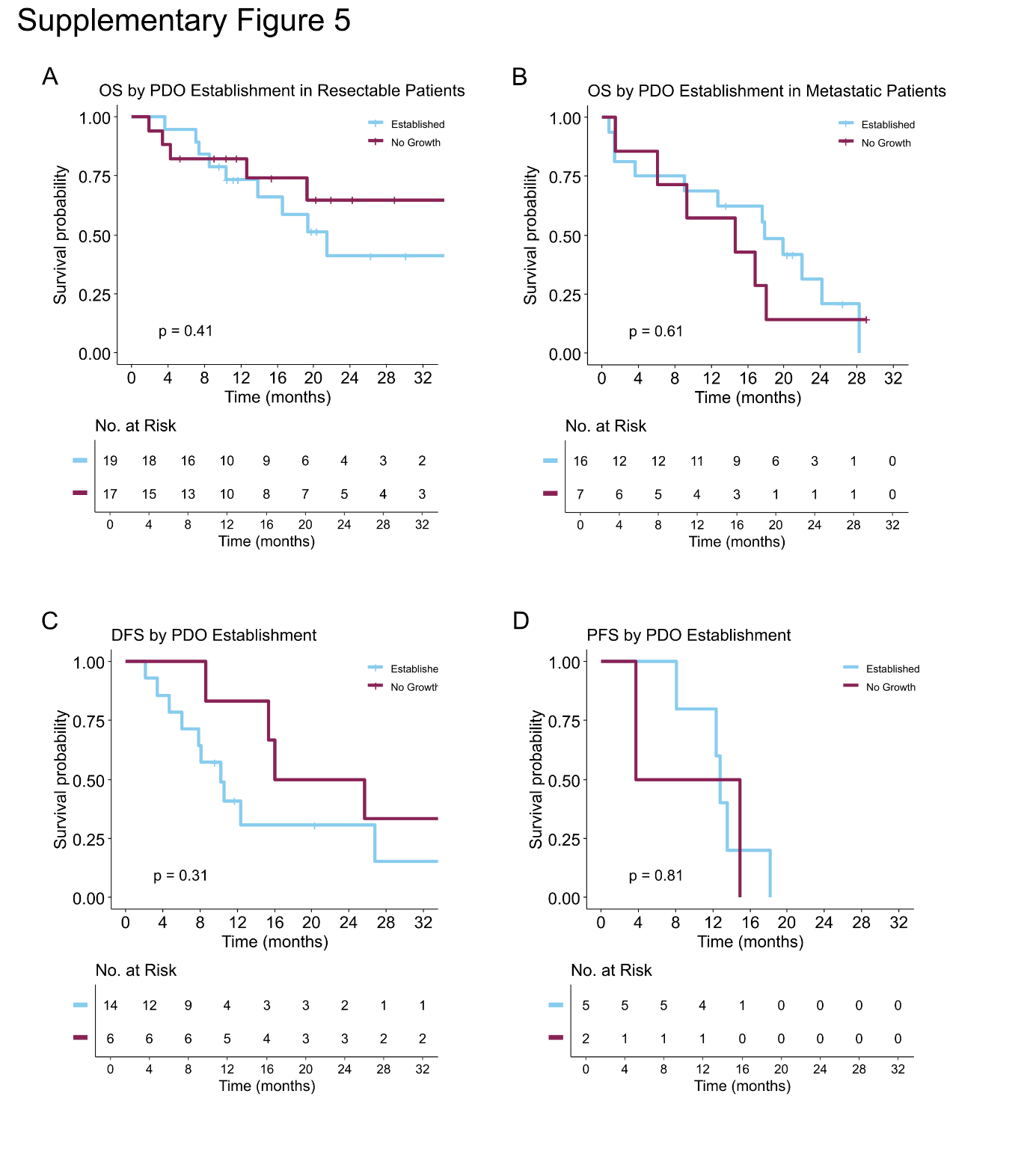
***

***Supplementary Figure 5: Kaplan Meier Curves of DFS and PFS depending on PDO establishment***

***A*** *OS in resectable patients was not correlated with PDO growth.* ***B*** *OS in metastatic patients was not correlated with PDO growth.* ***C*** *A trend towards longer DFS was observed in resectable patients.* ***D*** *PFS was not correlated with PDO growth in metastatic patients.*
